# Individual characteristics associated with physical activity intervention delivery mode preferences among adults

**DOI:** 10.1186/1479-5868-11-25

**Published:** 2014-02-25

**Authors:** Camille E Short, Corneel Vandelanotte, Mitch J Duncan

**Affiliations:** 1 School of Human, Health and Social Sciences, Centre for Physical activity studies, Central Queensland University, Rockhampton, Australia

## Abstract

**Background:**

People have different preferences on how health behaviour change interventions are delivered to them; intervention implementation, retention and effectiveness may be improved if preferences can be matched.

**Purpose:**

This study aims to explore factors related to preference of face-to-face, and group-, print- or web-based physical activity intervention delivery modes among adults recruited from the general population.

**Methods:**

A question relating to physical activity intervention preference was included in the telephone administered 2010 Queensland Social Survey. Multinomial regression models were used to explore socio-demographic (e.g., age, marital status, location), health (e.g., BMI, chronic disease status) and behavioral factors (e.g., internet use, physical activity, diet, social networking) related to intervention preferences, using ‘a face-to-face intervention’ as the reference category.

**Results:**

35.2% of those approached took part in the telephone interviews (n = 1,261). Preference for a web-based intervention was positively associated with being in the 35–44 age group (compared to the 18–34 age group; RR = 2.71), living in a rural area (RR = 2.01), and high internet use (RR = 1.03); and negatively associated with female gender (RR = 0.52), obesity (RR = 0.42), and higher physical activity participation (RR = 0.99). Preference for a print-based intervention was positively associated with older age (RR = 5.50); and negatively associated with female gender (RR = 0.48) and obesity (RR = 0.47). Preference for a group-based program was positively associated with living in a regional town (RR = 1.48) and negatively associated with being separated (RR = 0.45) and obesity (RR =0.56).

**Conclusion:**

Findings from this study help to delineate what physical activity intervention delivery modes are likely to be appealing for specific target groups, especially in relation to people of different weight status, age, gender and living environment. As such, this information will be useful in the development of interventions targeted at these groups.

## Background

Large proportions of people living in western societies do not meet public health guidelines for physical activity [1-4]. This is a major public health concern, due to the health issues associated with physical inactivity and the health benefits that participating in regular physical activity confers [5]. The challenge is to develop physical activity interventions that are appealing, effective and can be sustainably implemented at a population level. Obtaining a greater understanding of people’s preferences for how interventions are delivered can help inform this process by guiding development and implementation decisions, since efforts to promote physical activity may be more effective if intervention approaches are matched to the needs and interests of the target group [6,7].

Common physical activity intervention delivery modalities include face-to-face counselling, group-sessions, and mediated approaches such as telephone, information-technology, print-based and mass-media interventions. These intervention types differ in effect-size, reach, and maintenance, and thus in their potential for public health impact [8]. Face-to-face interventions involve education and counselling conducted during a one-on-one session in a pre-negotiated location. These interventions can be tailored to the specific needs and abilities of clients [8]. Group-based interventions are conducted in a group-setting, where participants can interact with each other and the group leader. These interventions are often not adapted to individual’s needs but rather encourage behaviour change by increasing social support [8]. Whilst both of these intervention types can be highly efficacious, reach and maintenance can be poor due to the high cost of delivery and barriers associated with participation [8]. In contrast, mediated interventions, such as those delivered via the telephone, information-technology and print-media are able to reach large numbers and can be disseminated at a relatively low cost [9]. Like face-to-face approaches, they can offer advice and information that is tailored to individual characteristics (when using automated expert systems) and depending on the modality can provide social support [9]. Although the effect-size of these interventions is often smaller than non-mediated approaches, the potential to impact public health may be greater given the ability to sustainably deliver the interventions in a consistent manner to large segments of the population [8].

Research examining adults preferred intervention delivery mode is scarce [10]. The majority of research to date has been descriptive and conducted among specific sub-populations, such as chronic disease groups and post-menopausal women [11-15]. This research has generally shown that preference for face-to-face interventions (including one-on-one and group-based programs) compared to mediated interventions is high [11-15]. To our knowledge, only one study has examined intervention delivery mode preferences in the general population. Booth et al. [15] examined intervention delivery mode preferences among a large sample of Australian adults, stratified by age and gender. Overall, results from this study reflect those conducted in sub-populations, with face-to-face counselling from a health professional (38% of all respondents) and group-based sessions (31% of all respondents) being the most preferred sources of physical activity support compared to mediated interventions. The stratification of study findings by age and gender provides some insight into how preferences may differ based on individual characteristics, with more women and young people reporting a preference for group-based support and more men and older people reporting a preference for face-to-face advice. However, this study was conducted more than 15 years ago and did not assess preferences for computer-mediated interventions. As such, the findings may not be representative of the general adult population in today’s society where there is increased access to and use of information technology [16]. Further, information regarding how individual characteristics, beyond gender and age, may influence intervention delivery mode preference is lacking. Given that intervention implementation, retention and effectiveness may be improved if intervention preferences can be matched, understanding who is and who isn’t interested in specific approaches is important.

This study aims to build on previous research by examining the physical activity intervention delivery mode preferences of adults and by exploring the association between preferred intervention delivery mode and important individual characteristics, including demographic, health and behavioral factors among a large sample of adults recruited form the general Australian population.

## Method

### Participants and procedure

The cross-sectional data analysed in this study was collected during July and August 2010 as part of the Queensland Social Survey, conducted by the Population Research Laboratory at the Central Queensland University in Australia. The survey was conducted by trained interviewers using computer assisted telephone interview (CATI) software. The target population included persons 18 years of age or older who, at the time of the survey, were living in a dwelling unit in Queensland that could be contacted by direct-dialled, land-based telephone service. A single respondent within each household was selected using pre-established guidelines to ensure an equal yet random selection of male and female participants. The selection and interview protocol has been described in detail elsewhere [17].

All participants provided informed consent at the start of the CATI survey. Approval from the Human Research Ethics Committee of CQUniversity Australia was obtained prior to data collection.

### Measures

#### Intervention preference

Preference for intervention delivery mode was assessed using one item ‘Which type of physical activity program would you prefer the MOST?’ asking participants to select one response from six response options (‘a face to face program with an instructor’, ‘a group-based program where I interact with other participants’, ‘a program I can do using the telephone’, ‘a program I can do on my own using mailed and printed materials’, ‘a program I can do on my own using the internet’, ‘unsure’). Prior to asking the question, a brief introduction was provided by the CATI interviewers to explain why physical activity interventions are needed, regardless of whether or not it was applicable to them or whether or not they would ever take part in such an intervention.

#### Demographic factors

Measured demographic factors included: gender, age (categorised as ‘18-34’, ‘35-44’, ‘45-54’, ‘55 and older’), household income (dichotomised as ‘≤ $1000/week’, ‘> $1000/week), employment status (categorised as ‘paid job’, ‘no paid job’), employment type (categorised as ‘full-time’, part-time’, ‘casual’), number of hours typically worked per day, marital status (categorised as ‘married/defacto’, ‘separated/divorced’, ‘single/other’), level of education (categorised as ‘primary school’, ‘secondary school’, ‘further education’), years of schooling, having children under 18 years old living at home (no/yes), birthplace (dichotomised as ‘Australia’, ‘other’), and geographical location (‘city’, ‘regional town’, ‘rural area’).

#### Health behaviours

*Dietary behaviours* were assessed using two items ‘how many serves of vegetables do you eat on a usual day?’ and ‘how many serves of fruit do you eat on a usual day?’ Participants were classified as eating sufficient fruit and vegetables if they met the public health guidelines of eating 2 or more serves of fruit and 5 or more serves of vegetables on a typical day [18]. *Physical activity* was measured using the Active Australia Survey, which has demonstrated good validity in different population [19,20]. Questions included items on duration and frequency of walking and moderate and vigorous-intensity physical activity in the previous week. To be eligible for reporting, all activities had to be performed continuously for at least 10 minutes at a time. Total physical activity was calculated using this formula: total walking + moderate activity + (vigorous activity * 2). To meet the physical activity guideline, 150 minutes of activity a week over 5 days were needed [21,22]. *Smoking status* was assessed using one item ‘are you presently a smoker?’ (yes/no). *Sitting at work* was measured using one item ‘what do you estimate is the total time that you spend sitting during an average working day (recoded as ‘0-4 hours’, ‘>4-8 hours’, ‘>8-16 hours’) [23].

#### Health factors

Height and weight were self-reported in order to calculate body mass index (BMI; kg/m^2^). BMI was then used to classify participants as either ‘underweight’ (BMI <18.5), ‘acceptable weight’ (BMI 18.5-25), ‘overweight’ (BMI >25-30) or ‘obese’ (BMI >30). Chronic health issues were assessed using one item ‘have you ever been told by your doctor that you have any chronic health problems’ (yes/no). Global health was assessed using one item ‘would you say that your general health is’ with five response options (‘excellent’, ‘very good’, ‘good’, ‘fair’, ‘poor’).

#### Internet factors

*Internet access* and *use* were assessed using three items: *‘*Can you access the internet from your home computer?’ (yes/no), ‘What do you estimate was the total time that you spent using the internet for personal use in the last week?’, and ‘During the past 12 months how often have you used online based social networking sites such as Facebook, Myspace, Flickr, Twitter?’ (response options: ‘everyday’, ‘most days’, ‘once a week’, ‘once a month’, ‘less than once a month’, ‘never’). In addition, use of mediated approaches for communication with friends and family was assessed using one item ‘In general, how often do you have email, telephone or mail contact with friends or relatives not living with you? (responses options: ‘very often’, ‘often’, ‘sometimes’, ‘occasionally’, ‘rarely’, ‘never’).

### Analysis

Descriptive statistics were calculated for all variables and compared across intervention preference groups using chi-square tests (for categorical variables) and analyses of variance (for continuous data). Multinomial logistic regression models were conducted to model the likelihood of intervention preference choice relative to the reference category (‘a face-to-face program with an instructor’ served as the reference category in all analyses) as a function of demographic, behavioral, health and internet variables. ‘A face-to-face program with an instructor’ was chosen as the reference category because it is the most preferred intervention type among adults [11-15] and because it may be the least promising in terms of public health impact, due to the high cost associated with delivery and issues with reach and maintenance [24-26].

Univariate analyses were run with the regression of the dependent variable (preference for intervention delivery-mode) on each of the independent variables (potential correlate) using the whole sample. Only independent variables which had a p-value of 0.25 or less were selected for inclusion in the multinomial models [27]. Based on these analyses, income, internet access at home, self-rated health, years of schooling, work status, work hours and sitting at work were dropped from subsequent multivariate models. To screen for potential multicollinearity problems, a correlation matrix was produced examining the correlation between all remaining independent variables. If two variables were found to be strongly correlated with each other (i.e., >50%), [27] only one variable (i.e., the strongest predictor) was retained. Children under 18 years old living at home and use of social networking sites were found to be highly correlated with age (negative association), and were dropped from the analysis.

Separate models were conducted for demographics (model 1), health variables (model 2), and behavioural variables (including internet behaviors; model 3). Only variables demonstrating a significant relationship (p ≤ 0.05) with preference for delivery mode in these models were then entered into a combined model (model 4). Relative risks are reported to show the magnitude of association [27]. Stata version 11 (Stata-Corp) was used for all analyses.

## Results

### Participants

The overall response rate for the Queensland Social Survey was 35.2%. Of the 1261 participants, 1,137 (90%) were included in the analyses (60 cases were omitted from analysis due to missing response for preferred intervention delivery type, 36 cases were omitted due to membership in the ‘telephone’ or ‘not-sure’ intervention preference categories and 28 cases categorised as ‘underweight’ were omitted due to insufficient sample size in these groups). Participant characteristics are presented in Table 1. Overall, the majority of participants were married (73%), working full-time (66%), non-smokers (85%), overweight or obese (66%), had access to the internet at home (94%), and had insufficient vegetable intake (82%). A greater proportion of participants reported preferring face-to-face intervention delivery modes (group-based, 40%; one-on-one with an instructor, 33%) than mediated delivery modes (telephone, <1%; print, 10%; internet, 9%). There were some significant differences in the socio-demographic profiles between intervention preference groups. Differences between groups were found for participant age, gender, number of children under 18, BMI category, chronic illness status, number of hours spent on the internet per week for personal use and frequency of social media use (Table 1).

**Table 1:** Participant characteristics by intervention preference

	**Overall sample (n = 1261)**	**Face-to-face (n = 412; 36%)**	**Group (n = 499; 44%)**	**Print (n = 122; 11%)**	**Web (n = 104; 9%)**	**P**
**Demographics**						
Married/defacto (%)	73	72	75	81	69	0.06
Age	52.79 (16.31)	50.65 (15.94)	51.55 (16.21)	58.20 (14.81)	48.71 (15.04)	0.01
Male (%)	50	46	45	62	61	0.01
Further education (%)	53	58	51	54	63	0.06
Working Full-time (%)	66	64	65	69	71	0.62
Work hours	7.96 (2.48)	8.15 (2.37)	7.85 (2.61)	8.12 (2.66)	7.57 (2.25)	0.24
Children < 18 (%)	35	35	40	21	41	0.01
Location (%)						
City	52	55	49	52	50	0.22
Town	26	26	28	22.	23.	
Rural area	22	18	23	25	27	
**Health**						
BMI	30.03 (14.67)	31.13 (16.07)	29.30 (15.05)	28.23 (8.93)	28.51 (10.15)	0.09
Overweight%)	36	34	35	44	46	0.01
Obese (%)	30	35	27	26	24	
Chronic illness (%)	46	43	42	58	51	0.01
**Behaviour**						
Sufficient Physical activity (%)	57	60	57	54	49	0.29
Hours sitting at work	3.64 (3.09)	3.68 (3.03)	3.43 (3.08)	3.72 (3.11)	4.12 (3.39)	0.36
Sufficient fruit intake (%)	58	58	59	56	50	0.35
Sufficient vegetable intake (%)	18	15	18	21	19	0.48
Current smoker (%)	15	16	15	11	15	0.63
Mediated contact with family and friends (%)						0.02
Often	77	78	81	77	65	
Sometimes	18	18	15	19	31	
Rarely-Never	5	5	4	4	5	
**Internet access and use**						
home internet access (%)	94	94	94	91	97	0.38
Social media use (%)						0.01
Every day –most days	20	26	19	10	24	
Never	66	57	63	78	55	
Internet hours (personal use/last week)	6.60 (7.69)	7.29 (8.16)	6.03 (7.35)	5.38 (6.97)	8.98 (8.21)	0.01

### Factors associated with preferred delivery mode

Results for Multinomial logistic regression models 1–3 are presented in Table 2.

**Table 2:** Multinomial logistic regression results for the model 1–3 to determine associations with preference for intervention delivery mode

	**Group-based intervention**		**Print intervention**		**Web-based intervention**	
	***b *** **(95% CI)**	**RR**	***b *** **(95% CI)**	**RR**	***b *** **(95% CI)**	**RR**
**Model 1 - Demographics (n = 1148)**						
Marital status						
Married/defacto	1	.	1	.	1	.
Separated	−0.38 (−0.91; −0.15)	0.68	−0.29 (−1.08; 0.48)	0.74	0.72* (0.03; 1.42)	2.06
Single/other	−0.03 (−0.38; 0.32)	0.97	−0.47 (−1.14; 0.18)	0.62	0.19 (−0.41; 0.80)	1.21
Age						
18-34	1	.	1	.	1	.
35-44	0.34 (−0.12; 0.81)	1.41	0.26 (−0.78; 1.31)	1.30	0.99* (0.23; 1.77)	2.71
45-54	0.14 (−0.30; 0.58)	1.15	0.94* (0.03; 1.85)	2.57	0.42 (−0.36; 1.19)	1.52
55+	0.02 (−0.39; 0.46)	1.03	1.15** (0.27; 2.03)	3.05	0.00 (−0.78; 0.78)	1.00
Female gender	−0.02 (−0.29; 0.25)	0.98	−0.64** (−1.08; −0.21)	0.52	−0.65** (−1.10; −0.20)	0.52
Education level						
Primary	1	.	1		1	.
Secondary school	−0.65 (−1.56; 0.26)	0.52	−0.67 (−1.84; 0.48)	0.51	−0.45 (−2.09; 1.19)	0.64
Further education	−0.87 (−1.79; 0.04)	0.41	−0.64 (−1.81; 0.52)	0.53	−0.26 (−1.91; 1.37)	0.76
Unemployed		1.11	0.34 (−0.14; 0.81)	1.399	0.04 (−0.48; 0.56)	1.04
City	1		.	.	.	
Regional town	0.16 (−0.16; 0.47)	1.17	0.03 (−0.49; 0.54)	1.03	0.04 (−5.11; 0.58)	1.03
Rural area	0.31 (−0.03; 0.66)	1.37	0.30 (−0.21; 8.2)	1.35	0.57* (0.04; 1.11)	1.78
**Model 2 - Health variables (n = 1137)**						
Weight status						
Normal	1	.	1	.	1	.
Overweight	−0.20 (−0.52; 0.11)	0.81	0.23 (−0.25; 0.72)	1.26	0.28 (−0.23; 0.79)	1.33
Obese	−0.48** (−0.81; −0.15)	0.62	−0.37 (−0.91; 0.17)	0.69	−0.42 (−1.00; 0.16)	0.65
No chronic illness	0.02 (−0.24; 0.29)	1.02	−0.62** (−1.04; −0.22)	0.53	−0.34 (−0.77; 0.94)	0.71
**Model 3 - Behavioural variables (n = 837)**						
Physical activity (mins)	0.00 (−0.00; 0.00)	1.00	−0.00 (−0.00; 0.00)	0.99	−0.01* (−0.00; −0.00)	0.99
Sufficient veg	0.27 (−0.15; 0.68)	1.31	0.24 (−0.42; 0.89)	1.27	0.56 (−0.67; 1.19)	1.76
Sufficient fruit	−0.04 (−0.36; 0.28)	0.96	−0.10 (−0.61; 0.40)	0.89	−0.25 (−0.77; 0.25)	0.77
Internet use (hrs)	−0.02* (−0.04; −0.00)	0.97	−0.03 (−0.07; 0.00)	0.96	0.02 (−0.00; 0.04)	1.02
Mediated contact						
often	1	.	1	.	1	.
sometimes	−0.22 (−0.64; 0.21)	0.80	−0.29 (−0.99; 0.42)	0.75	**0.81 (0.25; 1.38)	2.26
Rarely/never	0.35 (−0.58; 1.31)	1.44	−0.11 (−1.71; 1.49)	0.89	0.79 (−0.48; 2.07)	2.21

Base category = ‘a face to face intervention with an instructor’.

*p < 0.05; **P < 0.01.

Model 1 found that being separated (*RR* = 2.06, *P* < 0.05), being within the 35–44 aged group (*RR* = 2.71, *P* < 0.05), and living in a rural area (*RR* = 1.77, *P* < 0.05) was associated with a greater likelihood of preferring a web-based intervention over a face-to-face intervention. Being older aged (55 + yrs old, RR = 3.15, *P* < 0.05) was associated with an increased likelihood of preferring a print-based intervention over a face-to-face intervention. Being female was found to decrease the likelihood of preferring a web-based intervention (*RR* = 0.52, *P* < 0.01) and a print-based intervention (*RR* = 0.52, *P* < 0.05). There were no significant demographic predictors for preferring a group-based intervention compared to a face-to-face intervention. The variance accounted for by the demographic model was 3% (Nagelkerke R^2^ = 0.03, *P* = < 0.01).

Model 2 found that obesity (*RR* = 0.62, *P* < 0.01) was associated with a decreased likelihood of preferring a group-based program over a face-to-face program, and that having no chronic health conditions (*RR* = 0.53, *P* < 0.01) was associated with a decreased likelihood of preferring a print-based intervention over a face-to-face program. There were no health predictors significantly related to preferring a web-based intervention over a face-to-face intervention. The variance accounted for by the health model was 1% (Nagelkerke R^2^ = 0.01, *P* = < 0.01).

Model 3 found that higher levels of total physical activity (RR = 0.99, *P* < 0.05) were associated with a decreased likelihood of preferring a web-based program over a face-to-face program. Occasional use of mediated sources (e.g., email, telephone) to contact family and friends (compared to very often, RR = 2.26, *P* < 0.01) was associated with increased likelihood of preferring a web-based program over a face-to-face program. Higher levels of internet usage in the last week was associated with a decreased preference for group-based programs (RR = 0.98, *P* < 0.05) compared to face-to-face programs. There were no significant behavioural predictors related to preferring a print-based intervention over a face-to-face intervention. The variance accounted for by the behavioural model was 2% (Nagelkerke R^2^ = 0.02, *P* = < 0.01).

The results of model 4 (the combined model) are reported in Table 3. The combined model found that after controlling for all factors, preference for the web-based intervention over a face-to-face intervention was positively associated with being in the 35–44 aged group (compared to the 18–34 age group), living in a rural area, and internet use; and negatively associated with female gender, obesity, and total physical activity participation. Preference for a print-based intervention over a face-to-face intervention was positively associated with older age; and negatively associated with female gender and obesity. Preference for a group-based program over a face-to-face program was positively associated with living in a regional town (compared to a city); and negatively associated with being separated and obesity. The variance accounted for by the combined model was 6% (Nagelkerke R^2^ = 0.06, *P* = < 0.01).

**Table 3:** Multinomial logistic regression results for model four (the combined model) to determine associations with preference for intervention delivery mode

	**Group-based intervention**		**Print intervention**		**Web-based intervention**	
	***b *** **(95% CI)**	**RR**	***b *** **(95% CI)**	**RR**	***b *** **(95% CI)**	**RR**
**Demographics**						
Marital status						
Married/defacto	1	.	1	.	1	.
Separated	−0.81* (−1.53; −0.09)	0.45	−1.40 (−2.91; 0.82)	0.24	0.74 (−0.08; 1.57)	2.10
Single/other	0.02 (−0.42; 0.47)	1.02	−0.31 (−1.15; 0.53)	0.73	0.24 (−0.54; 1.02)	1.26
Age						
18-34	1	.	1	.	1	.
35-44	0.47 (−0.07; 1.01)	1.59	0.86 (−0.41; 2.12)	2.35	0.99* (0.08; 1.91)	2.71
45-54	0.22 (−0.30; 0.75)	1.24	1.03 (−1.17; 2.22)	2.78	0.44 (−0.48; 1.37)	1.56
55+	0.37 (−0.14; 0.88)	1.44	1.70** (0.56; 2.85)	5.50	−0.02 (−0.97; 0.93)	0.98
Female gender	−0.18 (−0.50; 0.15)	0.84	−0.73** (−1.27; −1.86)	0.48	−0.64* (−1.19; −0.08)	0.52
Location						
City	1	.	1	.	1	.
Town	0.39* (0.02; 0.77)	1.48	−0.01 (−0.64; 0.66)	1.01	0.38 (−0.24; 1.02)	1.47
Rural area	0.37 (−0.06; 0.81)	1.45	0.46 (−0.19; 1.12)	1.58	0.70* (0.02; 1.37)	2.01
**Health variables**						
Weight status						
Normal	1	.	1	.	1	.
Overweight	−0.20 (−0.59; 0.18)	0.81	−0.20 (−0.81; 0.41)	0.82	0.36 (−0.27; 0.97)	1.42
Obese	−0.56** (−0.97; 10.16)	0.56	−0.75* (−1.4; −0.06)	0.47	−0.86* (−1.63; −0.08)	0.42
No chronic illness	0.08 (−0.26; 0.43)	1.09	−0.25 (−0.80; 0.29)	0.77	−0.42 (−0.98; 0.14)	0.65
**Behavioural variables**						
Total physical activity (mins)	−0.01 (−0.01; 0.01)	0.99	−0.01* (−0.01; −0.00)	0.99	−0.01* (−0.01; 0.00)	0.99
Internet use (hrs)	−0.02 (−0.04; 0.01)	0.98	−0.02 (−0.06; 0.02)	0.97	0.03* (0.01; 0.06)	1.03
Mediated contact						
often	1	.	1	.	1	.
Sometimes	−0.22 (−0.66; 0.22)	0.80	−0.35 (−1.09; 0.39)	0.70	0.59 (−0.02; 1.19)	1.79
Rarely/never	0.39 (−0.58; 1.34)	1.47	−0.28 (−1.92; 1.37)	0.76	0.58 (−0.75; 1.92)	1.79

Base category = ‘a face to face intervention with an instructor’, n = 814.

*p < 0.05; **P < 0.01.

## Discussion

Mediated intervention approaches have been put forth as promising public health approaches for physical activity promotion due to their potential for wide reach, accessibility, effectiveness and cost-effectiveness [28-30]. However, results from the current study suggest that the desirability of these interventions among the population, especially for telephone-based interventions is low and rather face-to-face physical activity programs (group-based or one-on-one with an instructor) that are more resource intensive to deliver are preferred. Given the need for more sustainable behaviour change approaches this is of concern and may represent a significant public health challenge. Whilst several reviews have shown that mediated interventions can be effective [28-30], information relating to participant representativeness and the reach, adoption and maintenance of the implemented interventions is scarce [31]. As such, it is difficult to determine if these interventions will be effective in a real world-setting, where preference for them is low. In order to boost the public health impact of mediated interventions it may be necessary to combine them with other more preferred approaches, (which has been shown to enhance efficacy of telephone-based interventions [28]) and/or develop strategies to enhance their appeal. The results of this study provide some insight into what target groups may be likely to adhere to mediated intervention and what target groups may require additional encouragement or assistance to adhere to these approaches.

### Comparison to previous research

Our results support the previous research findings that men are more likely to prefer mediated (print and online) interventions than women [14] and that people with lower physical activity levels are more likely to prefer mediated programs than people with higher levels of physical activity [15]. The preference for mediated intervention among men may be due to men’s desire to self-monitor their health, maintain their regular activities, and to obtain health information independently and judge illness severity before seeking help [32]. There may also be social factors contributing to this preference, such as men’s traditional social roles (e.g., difficulty relinquishing control; immunity and immortality; perception that men are not interested in prevention; and lack of male care providers) [33]. Among those with lower physical activity levels, mediated interventions may be perceived as less confronting, and hence preferred to face-to-face interventions. This seems plausible, since low activity levels are associated with lower self-efficacy, knowledge and skills [34].

The finding that having a chronic disease is associated with an increased preference for print-based interventions (in the health model) in the current study is in contrast to studies exploring intervention preferences among individuals with diabetes [12] and cancer [13]. In both of these studies, a strong preference for face-to-face interventions over mediated interventions (telephone, video-tape, pamphlet, and internet) was reported. One explanation for the different findings observed in the current study is that age may have had a confounding effect, since older age is associated with both the preference for print-based interventions and the likelihood of having a chronic disease [35,36]. Alternatively, it may be that the distinct etymology associated with different chronic diseases impacts on an individual’s physical activity support needs. In the case of elderly people, a wide range of barriers associated with older age, such as lack of transportation facilities, financial considerations, lack of affiliation to the fitness center culture, or social embarrassment, may contribute to a preference for mediated interventions that are home-based [37].

To our knowledge, previous research has not examined the influence of geographical location, weight status or internet use on preference for intervention delivery mode. In doing so, we found some novel findings. Namely, living in a regional town was associated with preferring group-based interventions and living rurally was associated with preferring online interventions. This may be due to the unique social structures and availability of health services present in each of these settings [38,39]. For example, individuals that live in rural Australia face transport and accessibility problems that are not experienced by those living in regional and urban areas [39], potentially making the practicality of online interventions more attractive. The interactivity of these interventions may also enhance their appeal, since social isolation is also often experienced in rural settings [38,40]. Another novel finding was that obesity was strongly associated with preferring face-to-face interventions with an instructor over any other delivery mode. Possible reasons for this include being too shy or embarrassed to exercise in front of a group and/or a perceived need for greater support than what they believe could be provided via a mediated intervention [41]. This could have public health implications, given the health and economic burden associated with obesity [42,43] and the need for sustainable approaches. Finally, this study also found that being in the 35–44 age group, compared to being in the 18–34 age group, was associated with a greater preference for web-based interventions over face-to-face interventions. This may reflect the increasing use of the internet among this age group at work (51%), and at home (52% access internet at home daily) [44], and/or the difficulty or perceived difficulty of attending face-to-face sessions at this stage of life due to a busy social or professional life [45].

### Strengths and limitations

The major strength of this study is that it is the first to examine the relationship between physical activity intervention delivery mode preference and individual characteristics among adults. A second strength is that the research was conducted in a large sample recruited from the general population. Research in this field is scare and is predominantly limited to research describing intervention preferences in special sub-population groups. Hence, this study has contributed to the literature by expanding current knowledge about adults’ physical activity intervention delivery mode preferences and by highlighting what factors may influence these choices.

This study also has some limitations that should be considered when interpreting results. First, there was over-sampling of older adults and under sampling of younger adults. This is potentially due to the use of landline only sampling. Whilst recent studies have shown that the exclusion of mobile only households does not (yet) significantly influence survey results [46], it is possible that this sampling method led to response bias, since younger people increasingly live in a mobile only household [47]. Further, due to the under-sampling of adults aged 18–24 and 25–34 years it was necessary to combine these age groups in order to perform the analyses. Given age-related differences in several factors that may influence intervention preferences (e.g., stage of life and lifestyle) it is possible that examining these two age categories separately would yield different results. This will require further analysis. Second, the data for this study was collected in 2010. More recent data may yield different outcomes, given the rapidly changing telecommunications environment. However, our review of previous research suggests that our data reflects current intervention preference trends [10,11]. Third, the individual characteristics explored in this study were not exhaustive. Although our study investigated a range of potential demographic, health and behavioural correlates of preference for intervention delivery mode, other potential correlates such as psychosocial factors were not explored. This may account for the small proportion (6%) of variance explained by the factors included in the combined model, since psychosocial factors typically explain 30-40% of the variance in physical activity behaviour [48-50]. Psychological factors that may be relevant to assess in future studies could include perceived physical activity barriers, preference for physical activity in groups or alone, social support and the importance placed on social interaction, as well as how each of these interacts with key socio-demographic, environmental and health factors [51]. Finally, due to the low proportion (<1%) of people who reported a preference for telephone based counselling these were excluded from the analysis and thus we were unable to explore preference characteristics related to this delivery mode which is still currently used in research settings and by Government agencies.

### Implications for practice

Despite accounting for only a small proportion of variance, the results may have implications for practice as they suggest that certain population groups, such as males and females, the young and the old, those living in regional and rural areas and those with a high body mass index may be responsive to different interventions types. However, the results also demonstrate that the preference for mediated interventions is low at a population level, especially among women, the obese, and those living in urban areas. If we are to successfully reduce the public health burden associated with physical inactivity, an increased effort to promote the desirability and accessibility of mediated approaches among these groups may be needed. Furthermore, attempts to adapt intervention strategies to the local context and target them to well-defined groups or individual characteristics may also further enhance efficacy [52]. This study helps delineate these groups and provides some insight into what approach may be most appealing to whom.

## Conclusion

In conclusion, our study reveals that demographic, health and behavioural variables have an influence on the preference for intervention delivery mode. Researchers and practitioners should consider these factors, when designing physical activity programs targeted at specific population groups. Further research examining these factors in combination with psychosocial factors may provide further insights.

## Competing interests

The authors declare that they have no competing interests.

## Authors’ contributions

CS was involved in conceptualisation, data analyses and manuscript development; CV was involved in conceptualisation, study coordination and manuscript development; MJD was involved in conceptualisation, data analyses and manuscript development. All authors read and approved the final manuscript.
